# Progress in Synthesis of Conductive Polymer Poly(3,4-Ethylenedioxythiophene)

**DOI:** 10.3389/fchem.2021.803509

**Published:** 2021-12-24

**Authors:** Shisong Nie, Zaifang Li, Yuyuan Yao, Yingzhi Jin

**Affiliations:** ^1^ China-Australia Institute for Advanced Materials and Manufacturing, Jiaxing University, Jiaxing, China; ^2^ National Engineering Lab of Textile Fiber Materials and Processing Technology (Zhejiang), Zhejiang Sci-Tech University, Hangzhou, China

**Keywords:** conducting polymer, PEDOT, doping, synthesis method, conductivity

## Abstract

PEDOT is the most popularly used conductive polymer due to its high conductivity, good physical and chemical stability, excellent optical transparency, and the capabilities of easy doping and solution processing. Based on the advantages above, PEDOT has been widely used in various devices for energy conversion and storage, and bio-sensing. The synthesis method of PEDOT is very important as it brings different properties which determine its applications. In this mini review, we begin with a brief overview of recent researches in PEDOT. Then, the synthesis methods of PEDOT are summarized in detail, including chemical polymerization, electrochemical polymerization, and transition metal-mediated coupling polymerization. Finally, research directions in acquiring high-quality PEDOT are discussed and proposed.

## 1 Introduction

Conductive polymer (CP) was discovered by Hideki Shirakawa, Alan Heeger, and Alan MacDiarmid in 1977 (Shirakawa et al., 1977; Park et al., 1980; Park, 1988). They demonstrated that the conductivity of polyacetylene can be adjusted over a few orders of magnitude. The CPs not only share similar electrical properties with metal or semiconductor, but also have good mechanical properties like polymer (Heeger, 2001). Therefore, CPs have attracted wide attention in the printable electronics, energy conversion and storage devices, biological electronics and so on (Guimard et al., 2007; Rozlosnik, 2009; da Silva and de Torresi, 2019; Jiang et al., 2020). Up to now, four CPs comprising polyacetylene (PA), polyaniline (PANi), polypyrrole (PPy), and polythiophene (PTh) have been reported and studied intensively. Although CPs have decent conductivity and flexibility, the poor stability caused by the doping state and insufficient half-life of conductivity pose an important challenge to the commercialization of CPs (Kudoh et al., 1999). The problem was not solved until the invention of Poly (3, 4-ethylenedioxythiophene) (PEDOT) in the late 1980s (Bayer, 1988).

Among CPs, PEDOT has drawn most of the attention in both academic and industrial communities due to its relatively high conductivity and remarkable stability in ambient conditions, as well as its potential to be transparent in the visible range (Bayer, 1988). A lot of works have been done to improve the conductivity of PEDOT, the highest conductivity of 6,259 S cm^−1^ for thin films and 8,797 S cm^−1^ for single crystals have been reported (Cho et al., 2014; Gueye et al., 2016). Conductivity is an important parameter for CPs, as it directly determines their applications. Meanwhile, the improvement of conductivity is mainly due to the crystallinity and doping of PEDOT, which is directly caused by the different synthesis methods. The polymerization process of PEDOT is complicated, as it involves many oxidants and additives, thus a slight change might produce great influence on the properties of the final products. Over the last decades, researchers have made huge progress in the synthesis of PEDOT, but precisely control its crystallinity to achieve superior performance is still a major challenge in the field of PEDOT synthesis. In order to fully understand the properties of PEDOT, conjugated oligomer of EDOT are attractive materials as a model system. Oligo-EDOT derivatives were synthesized and investigated. It was found that the properties of the product could be tuned by the oligomer length, end group, and monomer composition with fine control (Spicer et al., 2017). In addition to the conductivity, the biocompatibility and non-toxicity of PEDOT are also deeply concerned by researchers (da Silva et al., 2018; Meng et al., 2019; Dominguez-Alfaro et al., 2021). In recent studies, a new type of conductive polymer-based biosensor has been obtained through monomer modification and polymerization (da Silva et al., 2018; Meng et al., 2020; Ritzau-Reid et al., 2020). In this review, we summarized the most used synthesis methods of PEDOT and their development trends. The synthesis method and corresponding conductivity are reviewed and discussed. Finally, the challenges and research directions are proposed.

## 2 Synthesis Methods for PEDOT

**FIGURE1:**
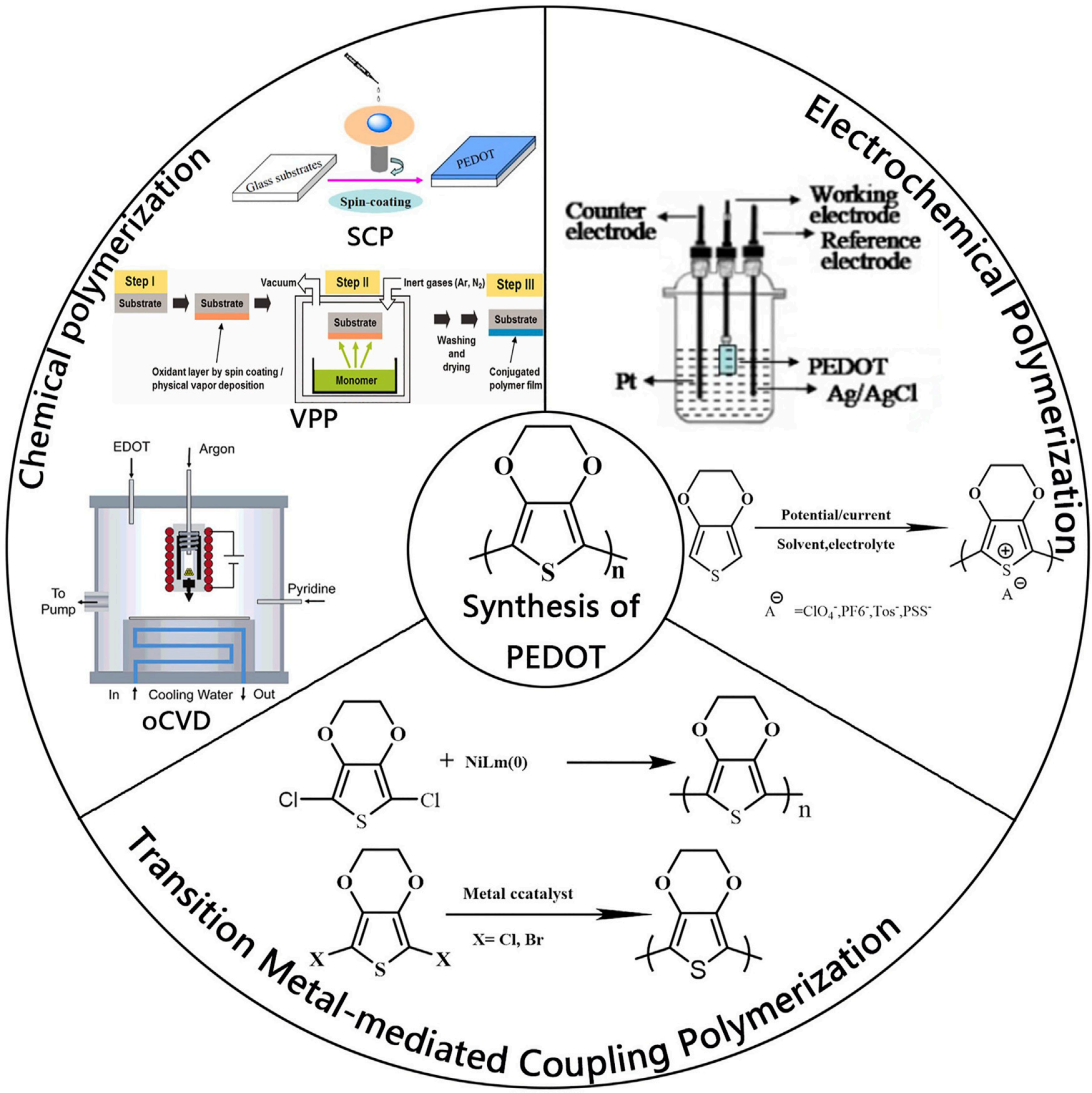
Three kinds of polymerization methods for PEDOT, chemical polymerization, electrochemical polymerization, and transition metal-mediated coupling polymerization. Reproduced with permission (Lock et al., 2006; Wen and Xu, 2017; Jiang et al., 2020).

The properties of PEDOT (optical transparency, electrical conductivity, work function) are highly dependent on the counterion and packing of PEDOT polymer. Electronic structure and optical absorption spectra of PEDOT for different oxidation levels have been studied using density functional theory (DFT) and time-dependent DFT (Zozoulenko et al., 2018). Therefore, the design and preparation of PEDOT with excellent performance are critical to realizing its wide application. Initially, PEDOT was prepared by oxidative polymerization of 3, 4-ethylenedioxythiophene (EDOT). So far, the polymerization methods of PEDOT can be classified into three categories (Figure 1): 1) chemical polymerization; 2) electrochemical polymerization; 3) transition metal-mediated coupling polymerization.

### 2.1 Chemical Polymerization

**FIGURE2:**
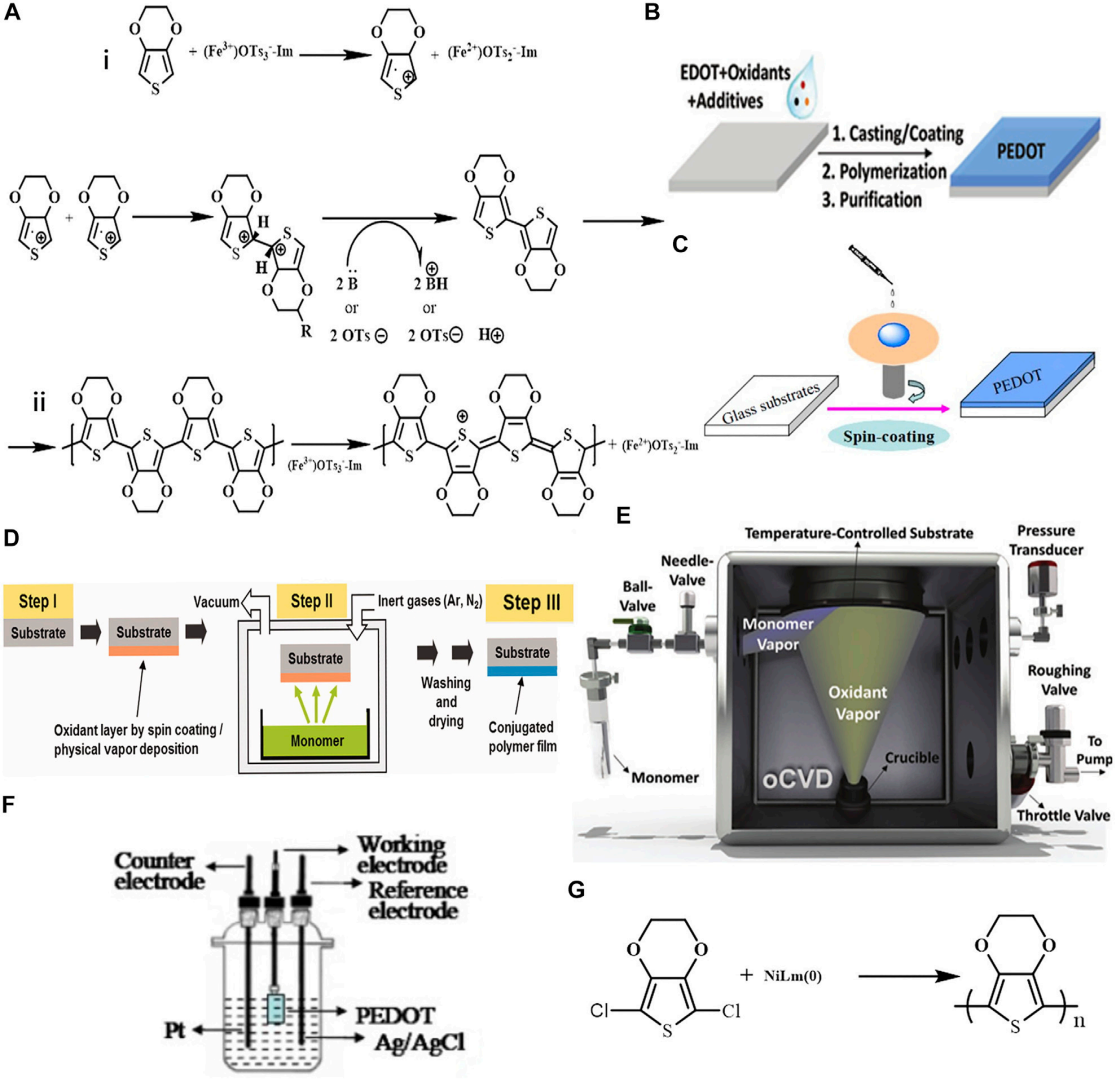
**(A)** schematic diagram of oxidative polymerization mechanism of PEDOT; **(B,C)** PEDOT *in-situ* solution polymerization process; **(D)** flow chart of vapor-phase polymerization; **(E)** schematic diagram of oCVD reactor; **(F)** a three-electrode device for electrochemical synthesis; **(G)** PEDOT is obtained by coupling polymerization of transition metals. Reproduced with permission (Ha et al., 2004; Bhattacharyya et al., 2012; Wen and Xu, 2017; Gharahcheshmeh and Gleason, 2018; Jiang et al., 2020).

Chemical polymerization is the most basic and commonly used method for the synthesis of PEDOT. With continuous development, this method has become the main method for the preparation of PEDOT and its derivatives. The oxidative polymerization mechanism of PEDOT can be divided into two steps (Figure 2A) (Ha et al., 2004). First, the EDOT monomer is oxidized to form cationic radicals followed by free radicals dimerization. The achieved dimer consequently experiences a deprotonation process, resulting in an active neutral dimer, which facilitates the dimer to react in the following oxidation process for chain growth. The neutral PEDOT is doped by the oxidants, anions of the oxidants are acted as counterions to stabilize the charged PEDOT (Jiang et al., 2020). The oxidants are usually utilized in the chemical polymerization process. However, PEDOT can also be prepared without oxidant by acid-assisted-polycondensation or self-polymerization of EDOT (Yin et al., 2013; Tomšík et al., 2020). Oxidative chemical polymerization is divided into oxidative polymerization of PEDOT dispersion and *in-situ* chemical polymerization according to the different usage of products. As for the above two methods, CPs with high conductivity can be obtained since the oxidant can simultaneously dope the conjugated PEDOT during the reaction process.

#### 2.1.1 Oxidative Chemical Polymerization of PEDOT Dispersion

The synthesis of PEDOT by oxidative chemical polymerization method is similar to the preparation of PPy. In this method, the iron (III)-chloride was introduced as the oxidant, resulting in insoluble PEDOT powders with a high conductivity (Heywang and Jonas, 1992; Jiang et al., 2012). Furthermore, metal ions Cerium (IV) (Corradi and Armes, 1997), Manganese (IV) (Hupe et al., 1995), and Cooper (II) (Im et al., 2008) have also been used as the oxidant to synthesize PEDOT. A breakthrough in preparing highly conductive PEDOT films was achieved after using iron (III)-sulfonates as oxidants (Jonas et al., 1990). The sulfonates are soluble in common organic solvents like ethanol or n-butanol, thus the PEDOT dispersions could be obtained through mixing EDOT and Fe (III)-sulfonates in these solvents. PEDOT with different microstructure could be synthesized by adjusting the polymerization method. Manohar et al., developed a reverse emulsion polymerization method, where sodium bis(2-ethylhexyl) sulfosuccinate and FeCl_3_ were used as the template and oxidant, to obtain PEDOT nanotubes with tube diameters in the range of 50–100 nm (Zhang et al., 2006). Zhang and collaborators proposed a facile soft-template-assisted self-assembled method to prepare PEDOT nanofibers and nanocubes with controlled morphology and size by simply adding solvent and tuning the ratio of monomer and solvent (Sun et al., 2017). Further research demonstrated that the addition of organic bases as an inhibitor in the reaction mixture could not only reduce the activity of the oxidant and thus slow down the polymerization rate, but also adjust the PH of the reaction, thus improving the pot-life and conductivity at the same time (De Leeuw et al., 1994; Winther-Jensen et al., 2005). These observations were mainly attributed to the formation of toluene sulfonic acid and its anions function as the counterions for the positively charged PEDOT. In addition, the acidity of the reaction solution is also regulated by the added inhibitors. The conductivity of PEDOT is also highly related to the reaction temperature and time. In the process of gas phase polymerization, the introduction of monomers and additives is closely related to the ambient temperature of the reaction. Water vapor as a proton cleaner plays a very important role in conductivity, while the removal of protons is directly determined by temperature (Goktas et al., 2015). Peroxides such as hydrogen peroxide, alkyl hydroperoxides, and diacyl peroxides can also be used as the alternative oxidants to prepare highly conductive PEDOT (Jiang et al., 2012).

#### 2.1.2 *In-Situ* Chemical Polymerization of PEDOT

*In-situ* polymerization refers to the oxidative chemical polymerization of EDOT directly on the substrates under film-forming conditions. PEDOT with regular molecular structure and high conductivity could be obtained by *in-situ* polymerization (Cho et al., 2014). The reaction mechanism of *in-situ* polymerization is roughly parallel to the oxidative polymerization (Fichou, 2008). Generally, the *in-situ* oxidative polymerization is preferably performed with iron (III), Manganese (IV), or other metal ions with a suitable higher oxidation state. The alcohol-soluble iron salts of sulfonic acids are ideal oxidants due to the limited solubility of EDOT in water. The p-toluenesulfonate has been taken as a very suitable anion and the corresponding iron (III) toluenesulfonate has become the most widely used oxidant in the preparation of PEDOT through *in-situ* method (Hong et al., 2005). Other metal salt oxidants and peroxides are also effective alternatives.

##### Solution-Cast Polymerization of PEDOT

There are three types of *in-situ* polymerizations: solution-cast polymerization (SCP), vapor phase polymerization (VPP), and oxidative chemical vapor deposition (oCVD). SCP is the simplest method for *in-situ* polymerizations of PEDOT, which was first reported by Bayer AG (Jonas et al., 1988). A typical procedure is performed as follows: the EDOT and oxidant were firstly dissolved in alcohol, then the mixture was cast onto the target substrate, followed by an annealing treatment to assist the polymerization. Finally, substrates were thoroughly rinsed to remove excess reagents (Figures 2B,C) (Gueye et al., 2020).

Kinetic studies show that the polymerization of PEDOT is determined by the slowest step of reaction rate, while the rate of EDOT monomer being oxidized by oxidant to generate free radicals is the slowest (Ha et al., 2004; Kim and Zozoulenko, 2019). Therefore, the oxidant plays a very important role in polymerization. In particular, the solubility, oxidation strength and stability of the oxidant have an important influence on the polymerization process. Anions in oxidants such as Cl^−^, tosylate (Tos^−^) and sulfonates also play important roles in the polymerization of PEDOT. These anions can not only neutralize the charge and further stabilize PEDOT (Brooke et al., 2017), but also affect the reaction rate, the molecular arrangement, as well as conductivity of PEDOT (Winther-Jensen et al., 2008). For iron-based oxidants, the standard electrode potential of cation reduction is constant, but the different anions can adjust the polymerization rate. Compared with Cl^−^, tosylate (Tos^−^) leads to lower effective oxidation strength of the oxidant. The polymerization rate of PEDOT can be reduced by iron p-toluenesulfonic acid (III) (Fe(Tos)_3_), which leads to a longer conjugated chain and smoother microstructure of PEDOT. Moreover, the conductivity of PEDOT is improved (Brooke et al., 2017). The polymerization rate is also related to the concentration of the oxidant. The PEDOT films obtained under low oxidant concentration exhibit higher conductivity and lower roughness. However, a challenge remains to prepare PEDOT films with conductivity over 1,000 S cm^−1^ by optimizing oxidants. There are two reasons that restrict the improvement of the conductivity of PEDOT. One is the influence of protons, which can accelerate the polymerization rate and decrease the conductivity. The generation of protons is inevitable, as shown in Figure 2A. The other is the side reaction caused by protons, which makes a large number of EDOT free radicals react into dimers or trimers, hindering the formation of long-chain PEDOT. The alkaline inhibitor as an additive was first proposed by De Leeuw et al. (1994). Imidazole was utilized as an alkaline inhibitor in the synthesis of PEDOT, which can increase the polymer chains by slowing down the reaction rate and restricting excessive doping. The PEDOT film achieved a high conductivity of 500 S cm^−1^ (Ha et al., 2004). In addition to alkaline additives, surfactants and co-solvents are also used as additives to assist the synthesis of high conductivity PEDOT. The conductivity of synthesized PEDOT can be up to 3,000 S cm^−1^ with polymeric surfactant poly(ethylene glycol)-block-poly(propylene glycol)-block-poly(ethylene glycol) (PEG-PPG-PEG) and high boiling point cosolvent (N-methyl-2-pyrro-lidone) as additives (Gueye et al., 2016).

##### Vapor-Phase Polymerization of PEDOT

VPP is another common synthesis method for *in-situ* polymerizations, in which reactants participate in the reaction under gas state. PEDOT films obtained by VPP showed excellent photoelectric properties (Rahman et al., 2011; Howden et al., 2013; Li et al., 2015; Brooke et al., 2017). VPP involves three steps (Figure 2D): 1) depositing a solvent containing an oxidant and/or additive on a substrate by solution processing method; 2) exposing the coated substrate to EDOT monomer vapor in a closed chamber for polymerization; 3) washing the deposited film to remove residual oxidant and adsorbed monomer (Bhattacharyya et al., 2012; Brooke et al., 2017). Oxidation reaction occurs at the interface between oxidants and monomers in gas phase. The PEDOT film-forming mechanism could be “bottom-up” when the oxidant mixture diffuses upward or “top-down” when the monomer diffuses downward, but this speculation is still debated (Jiang et al., 2020).

In VPP method, the problems brought by oxidants are the same as those in SCP, so it is necessary to use an inhibitor to control the reaction rate. Winther-Jensen and Keld West initially added pyridine as an alkali inhibitor in 2004. The use of pyridine can not only slow down the polymerization rate but also eliminate acidic side reactions. The conductivity of the finally obtained PEDOT film is improved, and the highest conductivity exceeds 1000 S cm^−1^ (Winther-Jensen and West, 2004). In terms of processing conditions, such as the pressure, temperature, and humidity in the reaction chamber directly affect the photoelectric properties of PEDOT. A highly ordered crystal structure can be obtained at 60°C, which significantly improves the conductivity (Kim et al., 2003). In terms of humidity, the existence of water is helpful to repeat the polymerization cycle, because it can be used as a proton scavenger on EDOT dimer (Fabretto et al., 2009; Mueller et al., 2012; Goktas et al., 2015). The conductivity of PEDOT was significantly improved, and the top surface of the film was smoother. However, the existence of water also brings serious problems to polymerization. Excessive load leads to the formation of microcrystals in the oxidant layer, which makes the oxidant inactive. Common oxidant salts (FeCl_3_ (Cho et al., 2014), Fe(Tos)_3_ (Bubnova et al., 2014; Fabretto et al., 2012), Fe(OTf)_3_ (Massonnet et al., 2015; Brooke et al., 2018)) showed high water affinity, which led to easy formation of hydrate crystallites. These crystalline regions are bad for the synthesized PEDOT and seriously affect the conductivity. To solve this problem, Zuber et al. used an amphiphilic copolymer inhibitor of PEG–PPG–PEG, which can inhibit the hydrate crystal growth of oxidant (Zuber et al., 2008). The most significant benefit of the VPP method is that the conductivity of the product is greatly improved. The conductivity of the synthesized single crystal PEDOT nanowires reaches the highest value at present, up to 8,797 S cm^−1^ (Cho et al., 2014).

##### Oxidative Chemical Vapor Deposition of PEDOT

The oCVD method is a one-step steam synthesis process in which oxidant and monomer meet on the target substrate in a steam state and undergo oxidative polymerization. This method can avoid the deposition of oxidants on the substrate, and the oxidant with good volatility can be selected. oCVD was initiated in 2006 (Lock et al., 2006) and fiber-shaped PEDOT with high conductivity was obtained. As shown in Figure 2E, to prevent the accumulation of oxidant, the substrate in the reactor is inverted above the oxidant crucible. Since the commonly used solid oxidants have low volatility, and oCVD requires oxidants to have certain volatility, the choice of oxidants is limited. Although Fe(Tos)_3_ is widely used in VPP, it cannot be used in oCVD because of its poor volatility. Therefore, metal halogen salts with good volatility (e.g., FeCl_3_ (Gharahcheshmeh and Gleason, 2018), CuCl_2_ (Im et al., 2008)) have become the mainstream in oCVD. In order to better control the concentration of the oxidant, liquid oxidants such as antimony pentoxide (SbCl_5_) (Nejati et al., 2014) and vanadium trioxide (VOCl_3_) (Nejati and Lau, 2011) have been developed for oCVD method in recent years. Compared with the solid oxidant, the surface concentration and flow rate of liquid oxidant VOCl_3_ can be conveniently adjusted during the reaction (Gharahcheshmeh and Gleason, 2018). The orientation of PEDOT (face-on and edge-on) has a strong correlation with the conductivity. Grissom and his colleagues used liquid and solid oxidants, respectively. Liquid oxidants can easily adjust the saturation ratio of oxidants (OSR). PEDOT obtained using liquid oxidants has face-on orientation, while PEDOT obtained using solid oxidants has edge-on orientation (Gharahcheshmeh et al., 2019). The PEDOT film with face-on orientation exhibits the highest in-plane electrical conductivity of 2,800 S cm^−1^ and the largest optical bandgap of 2.9 eV. In addition to liquid oxidants, volatile oxidants such as halogen gases have also been reported. The conductivity of the prepared Br-PEDOT film without post-treatment is 380 S cm^−1^ at 80°C, which is significantly higher than that of the PEDOT film obtained with ferric chloride as the oxidant at the same temperature, and the Br-PEDOT film is more stable (Chelawat et al., 2010). However, halogen gases have been shown to damage the apparatus through corrosion during the reaction. In VPP, the conductivity of PEDOT film can be greatly improved by using some additives. However, the lack of suitable volatile additives limits the further increase in conductivity in oCVD. Therefore, it is necessary to optimize the deposition parameters such as substrate temperature, pressure, and vapor flow to improve the performance of PEDOT films in oCVD. Ugur et al. (2015) reported that the grain size of PEDOT can be adjusted by adjusting the substrate temperature.

### 2.2 Electrochemical Polymerization

Electrochemical polymerization (Figure 2F) to prepare PEDOT was first demonstrated in 1988 (Heywang et al., 1988), and its concept is similar to that of oxidative chemical polymerization. The biggest difference with oxidative chemical polymerization is that no oxidants are used. In electro-polymerization, EDOT is oxidized by an applied potential and polymerization takes place at the electrode. Electro-polymerization requires a three-electrode system (counter electrode, reference electrode, and working electrode) and electrolyte solution. The electrolyte solution usually contains small molecules as electrolytes, and the commonly used electrolytes are lithium perchlorate (LiClO_4_), 1-butyl-3-methylimidazolium hexaphonate (BMIMPF_6_), and lithium bis (trifluoromethosulfonyl) amide (LiTFSI) (Zotti et al., 2003; Culebras et al., 2014). In the process of electro-polymerization, the anions of electrolyte are doped into PEDOT as counterions to stabilize the charge in PEDOT. Furthermore, anions greatly affect the morphologies, photoelectric properties, and mechanical properties of PEDOT films (Zotti et al., 2003; Poverenov et al., 2010; Culebras et al., 2014). Therefore, the electrolyte can be changed to introduce different counterions, and then the conductivity of PEDOT film can be adjusted. The maximum conductivity of PEDOT obtained by electro-polymerization is about 2,000 S cm^−1^ by adjusting electrolytes (Culebras et al., 2014). The doping and dedoping of PEDOT films can be realized by changing the applied potential and direction of the electro-polymerization reaction. Accordingly, the optoelectronic properties (e.g., ionic/electric conductivity, transparency, and Seebeck coefficient) can be tuned (Bubnova et al., 2012; Nguyen and Lee, 2016; Teran and Reynolds, 2017; van de Burgt et al., 2017; Jiang et al., 2020).

### 2.3 Transition Metal-Mediated Coupling Polymerization

Inspired by the role of transition metals in the coupling polymerization of thiophene polymers, Yamamoto et al. used transition metal nickel complexes to prepare neutral PEDOT using (Figure 2G) (Yamamoto and Abla, 1999). The PEDOT obtained by this method is in black, insoluble in water, and non-conductive. Therefore, this method is not widely used.

## 3 Conclusion and Outlook

PEDOT, as a very unique CP, possesses high conductivity, high environmental stability, high visible spectrum transparency, and multi-purpose processing ability, which makes it has great application potential in many fields. In this review, we mainly summarized the polymerization methods of PEDOT, such as chemical polymerization (E.g., SCP, VPP, oCVD), electrochemical polymerization, and transition metal-mediated coupling polymerization. The factors affecting the performance of PEDOT were briefly analyzed. The development of PEDOT conductivity under different polymerization methods was summarized. The existing problems and solutions of PEDOT under different polymerization methods were discussed. Although great progress has been made in the synthesis of PEDOT, there are still great challenges in the synthesis of high quality PEDOT. First, the conductivity of synthesized PEDOT is not high enough, and how to improve the conductivity of synthesized PEDOT to 10^4^ S cm^−1^ or even higher remains a challenge. Although many strategies have been used to improve the conductivity, the PEDOT cannot be mass-produced and the preparation conditions are harsh. In addition, the conductive mechanism of the synthesized PEDOT deserves further study to fundamentally optimize the conductivity of PEDOT. Finally, for different synthesis methods, we should develop new application directions and reach the full potential of PEDOT.
